# Electrochemical nanoimprinting of silicon

**DOI:** 10.1073/pnas.1820420116

**Published:** 2019-05-08

**Authors:** Aliaksandr Sharstniou, Stanislau Niauzorau, Placid M. Ferreira, Bruno P. Azeredo

**Affiliations:** ^a^The Polytechnic School, Arizona State University, Mesa, AZ 85212;; ^b^Department of Mechanical Science and Engineering, University of Illinois at Urbana–Champaign, Urbana, IL 61801

**Keywords:** nanoimprinting, metal-assisted chemical etching, silicon photonics, microfabrication, 3D silicon micromachining

## Abstract

Scalable nanomanufacturing enables the commercialization of nanotechnology, particularly in applications such as nanophotonics, silicon photonics, photovoltaics, and biosensing. Nanoimprinting lithography (NIL) was the first scalable process to introduce 3D nanopatterning of polymeric films. Despite efforts to extend NIL’s library of patternable media, imprinting of inorganic semiconductors has been plagued by concomitant generation of crystallography defects during imprinting. Here, we use an electrochemical nanoimprinting process—called Mac-Imprint—for directly patterning electronic-grade silicon with 3D microscale features. It is shown that stamps made of mesoporous metal catalysts allow for imprinting electronic-grade silicon without the concomitant generation of porous silicon damage while introducing mesoscale roughness. Unlike most NIL processes, Mac-Imprint does not rely on plastic deformation, and thus, it allows for replicating hard and brittle materials, such as silicon, from a reusable polymeric mold, which can be manufactured by almost any existing microfabrication technique.

## Significance

The indirect nature of existing parallel micromachining strategies that combine sacrificial templates with top-down processes to etch 3D micro- and nanostructures inherently produces poor out-of-plane patterning fidelity. Here, the patterning fidelity of our process is measured for microscale curvilinear 3D objects to be less than 20 nm in rms averaged over features as wide as 10 µm. These results are attributed to increased pathways for diffusion, which increase the kinetics of the anodic reaction. Using this approach, arrays of nanotextured silicon lenses are deterministically imprinted to illustrate Mac-Imprint’s ability to directly pattern hierarchical micro- and nanostructures and enable fabrication of biomimetic optical designs on silicon.

**Fig. 1.**
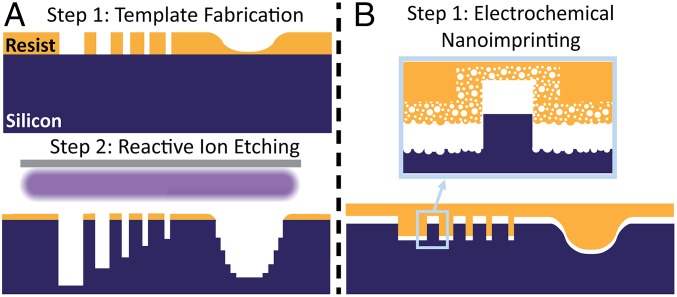
Schematics highlight (*A*) limitations of lithography and DRIE to fabricate hierarchical features, (*B*) the advantages of Mac-Imprint, and (*Inset*) its limitations on sidewall and bottom roughness.

Freeform 3D single-crystal silicon micro- and nanostructures offer the opportunity to topologically define the refractive index of a medium. In turn, they enable realization of a myriad of optical devices, such as classical elements (e.g., diffraction gratings), metasurface-based elements for silicon photonics (1, 2), X-ray flat lenses (3), optical resonators for biosensing (4), biomimetic imagers (5–7), and graded index materials (8) as antireflective and high-emissivity surfaces for photovoltaics and space applications (9), respectively. However, existing parallel bulk micromachining processes for silicon do not offer the ability to fabricate freeform structures with specific challenges when it comes to hierarchical micro- and nanoscale 3D features (10). At the root of this challenge is the indirect nature of existing parallel micromachining strategies that combine sacrificial templates—manufactured on a wafer-scale by either gray-scale lithography (11, 12), maskless lithography (13, 14), microstereolithography (15, 16), or nanoimprint lithography (17–19)—with top-down processes, such as deep reactive ion etching to etch 3D micro- and nanostructures (20). While this process combination led to sub–10-nm line resolution in planar memory devices, it cannot replicate such resolution out-of-plane due to (*i*) poor control over mask selectivity during etching, (*ii*) roughness produced via scalloping effects, and (*iii*) etch rate dependence on feature size in dry etching (Fig. 1*A*). In fact, this benchmark approach produces 3D features with poor surface finish (i.e., rms > 300 nm) and resolution in 3D (i.e., >2 μm), which are 100 times greater than in planar devices (21). In this context, in the early 2000s, Chou et al. (22) proposed direct and parallel patterning of silicon, which was subsequently expanded into mesoporous materials (23), metallic glasses (24), and crystalline metals (25–27) by the use of concomitant heating and mechanical imprinting or solid-state ionic stamping (27). A common drawback of heat-based imprinting processes is the limited control of the substrate’s crystal morphology. In the case of silicon, it produces area and line defects due to recrystallization. Recently, a room temperature catalyst-based wet etching technique—known as metal-assisted chemical etching (MACE) (28, 29)—was used in an imprinting configuration to pattern porous silicon—coined as Mac-Imprint (Fig. 1*B*)—to resolve the aforementioned fabrication challenges (30, 31). However, its implementations into nonporous silicon have been hindered by insufficient understanding of the modification of the reaction kinetics (32) and diffusion (33) mechanisms of MACE in the imprinting configuration, leading to high porosification of silicon concomitant with imprinting and condemning the optical and electronic properties of the substrate (34, 35).

**Fig. 2.**
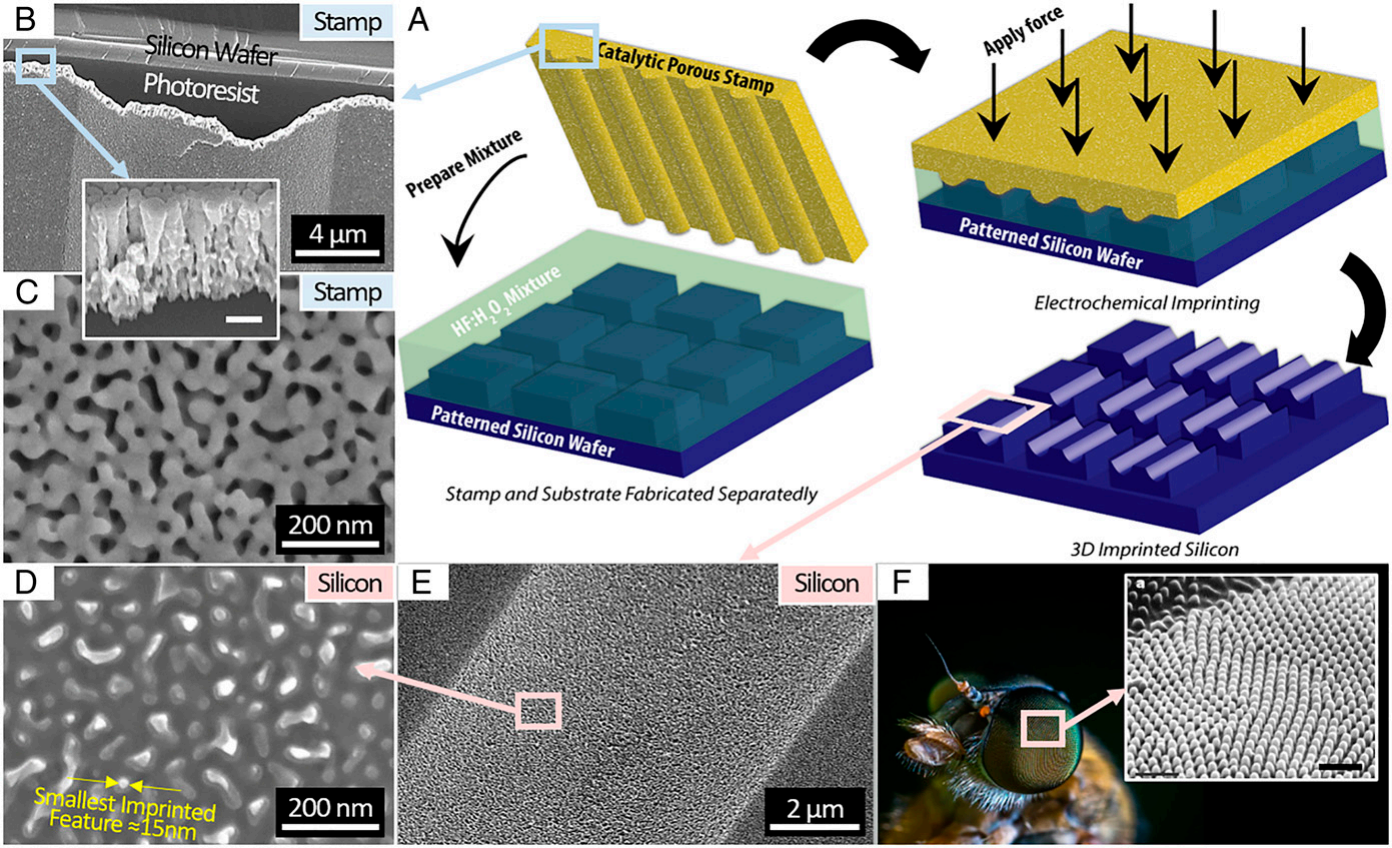
In *A*, the schematics show a Mac-Imprint stamp coated with a mesoporous Au film being used to electrochemically imprint an array of half-parabolic cylinders onto a prepatterned silicon substrate. In *B* and *C*, the SEM depicts the tilted (by 30°) cross-section and top-down views of the stamp, respectively, while *Inset* shows the cross-section detail of the mesoporous Au film. (Scale bar: *Inset*, 200 nm.) In *D* and *E*, the SEM depicts the top-down and tilted (by 30°) cross-section views of an imprinted parabolic half-cylinder, respectively. *D* highlights the reproduction of the mesoporous Au topology (*C*) onto the surface of the imprinted parabolic cylinder. In *F*, the image shows an insect’s compound eye in which the ommatidial surface and nipple array resemble the hierarchy of the imprinted silicon topology. (Scale bar: *Inset*, 1 µm.) Reprinted with permission from ref. 6.

At the core of Mac-Imprint is the use of a noble metal-coated stamp immersed in hydrofluoric acid (HF) and an oxidizer solution and brought in contact with an Si substrate to selectively induce etching of Si at the contact interfaces (as schematically illustrated in Fig. 2*A*). This process has (*i*) wafer-scale patterning capability and (*ii*) sub–20-nm shape accuracy in both vertical and horizontal directions. (*iii*) Stamps may be reused numerous times, and (*iv*) the Mac-Imprint process operates at pressures lower than 1 MPa (36). This process has (*v*) compatibility with roll-to-roll nanomanufacturing, (*vi*) low-cost tooling and earth-abundant consumables, and (*vii*) mesoscale bottom and sidewall roughness (Fig. 1*B*, *Inset*). Currently, Mac-Imprint is limited by (*i*) the lack of diffusion pathways to enable diffusion of reacting species (31), which significantly slows the etch rate, and (*ii* ) the lack of control of the kinetics of hole injection by the reduction of the oxidizer (30), which introduces porous defects into the silicon substrate. Several attempts to imprint silicon via MACE have repeatedly neither addressed these points nor examined the effect of imprinting conditions on the resulting morphology of imprinted substrates (30, 37–39), including (*i*) pattern transfer fidelity and (*ii*) porosity of imprinted substrates.

This paper presents two experiments performed with Mac-Imprint that elucidate the mechanism associated with each of those challenges. First, porosity is gradually introduced to the stamps by the synthesis of porous gold–silver thin-film catalyst (Fig. 2 *B* and *D*) with tunable porosity via timed dealloying, which has been well documented in literature (40). The catalyst porosity enables diffusion through the stamp and in turn, yields high pattern transfer fidelity (Fig. 2 *C* and *D*). The resulting morphology of imprinted silicon nanostructures and its porous defects are examined by optical and scanning electro microscopy (SEM), and they correlate directly to the stamp porosity. Second, the catalyst surface area in contact with solution is gradually varied relative to the contact area between stamp and substrate to demonstrate the scaling of kinetics of the cathodic reaction in Mac-Imprint with the former. The increase in the gold surface area modifies the kinetics of the cathodic reaction independent of the solution parameter, ρ, established by Chartier et al. (32). With these two elucidations, the barriers are reduced for electrochemical nanoimprinting of silicon implemented by the general scientific community. Finally, nanotextured parabolic half-cylinders were imprinted on silicon (Fig. 2 *D* and *E*) to demonstrate Mac-Imprint’s ability to pattern hierarchical micro- and nanoscale 3D features; such designs mimic the length scale and hierarchy of a moth’s eye and thus, illustrate Mac-Imprint’s versatility to manufacture complex biomimetic optical devices (Fig. 2*F*). Given the existing MACE literature on etching of III-V and II-VI semiconductors (41) and amorphous and polycrystalline silicon and germanium (42), it is plausible that these substrates could also be imprintable with this technique, extending the impact of this work.

**Fig. 3.**
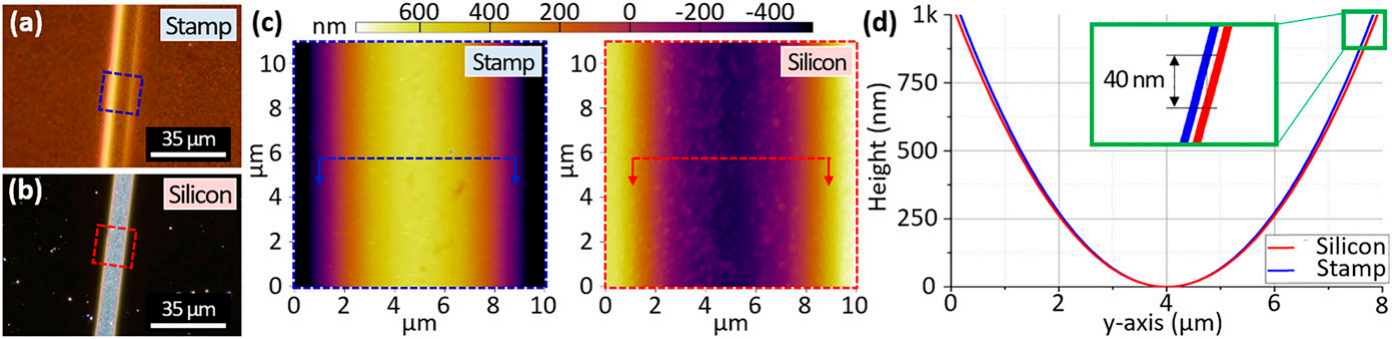
*A* and *B* are optical micrographs of the stamp and substrate after imprinting. In *C*, the topology of stamp (*Left*) and imprinted silicon substrate (*Right*) are measured via AFM at complimentary locations. Porous catalyst thin film with PVF (of 68%) was used in this case. In *D*, the cross-section profiles of stamp and substrate marked by color in *C* are superimposed to highlight accurate transfer of shape during imprinting.

Arrays of microscale parabolic half-cylinders (as depicted in Figs. 2*B* and 3*C*) were fabricated via lithography and resist thermal reflow onto a silicon wafer, and subsequently, a noble mesoporous thin film was synthesized containing pores with size distributions between 5 and 100 nm (Fig. 2*C*). With this selected geometry and fabrication approach, patterning fidelity (i.e., geometrical difference between stamp and substrate) for a wide range of 3D feature sizes could be measured. More complex stamp features have been previously demonstrated in refs. 30 and 31, and the technique extends, in theory, to any other stamp microfabrication approach in the literature provided that the stamp material selected is chemically resistant to the MACE’s solution selected.

**Fig. 4.**
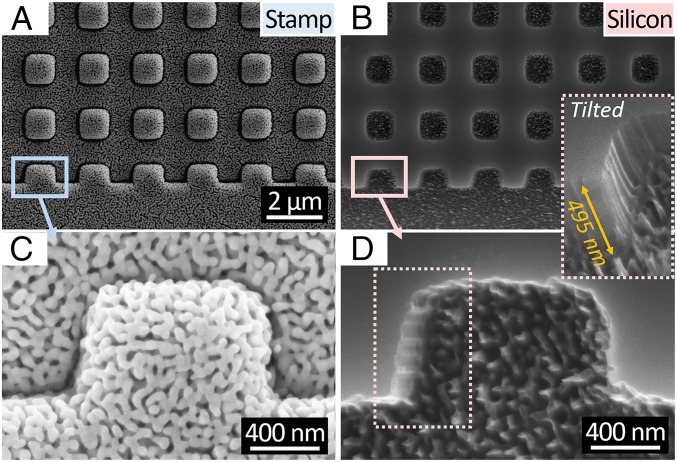
An array of pillars (*A* and *C*) patterned on porous Au with 1.07-µm width and 2-µm period and (*B* and *D*) corresponding imprinted Si. *D*, *Inset* (tilted by 52°) highlights the roughness induced at the bottom and sidewall of the feature due to the porous gold morphology of the stamp. Imprinting time was 2 min.

In Fig. 2*D*, it is shown that sub–15-nm features present in the stamp’s highly porous catalyst [i.e., with 75% apparent pore volume fraction (PVF)] are successfully imprinted onto the silicon substrate, demonstrating feature replication at the shortest length scale possible with MACE (43). Despite successful imprinting of the mesoporous stamp topology onto silicon (Fig. 2*D*), in the nanofabrication literature, resolution is typically reported as the line-width resolution of linear gratings. Thus, the line-width resolution of Mac-Imprint is limited by the largest pore size of the stamp, since porosity is a requirement. To highlight this limitation, a planar stamp composed of an array of square-shaped pillars with 1.07-µm width and 0.93-µm spacing was fabricated onto SU-8 thin film by nanoimprint lithography. Subsequently, such planar patterns were imprinted onto silicon to a depth of 0.50 µm. The roughness induced by the imprinting of the porous gold morphology (Fig. 1*B*) can be observed in the Si features, particularly at its bottom, in its edge contour and in its sidewall (Fig. 4). To remediate this effect, one could use straight-walled and ordered pores as the stamp (44) or reduce the pore sizes in the catalyst film into the microporous range by existing dealloying procedures (45). Also, this result highlights the ability of Mac-Imprint to produce straight-walled and high-aspect ratio 2D profiles within the etch depth limitations determined previously (31). Rounding of the feature’s corners is attributed to the stamp, which was sputtered by a 400-nm catalyst layer that effectively increased corner radius of curvature. In Fig. 3, the 3D pattern transfer fidelity between the stamp and substrate is measured by atomic force microscopy (AFM) at complimentary locations. It is found that, in the case of highly porous catalyst, microscale parabolic half-cylinders were imprinted into silicon and yielded sub–40-nm maximum shape deviation from the stamp across 10-µm-wide features (Fig. 3*D*). This small variation over such large length scale is attributed to the elastic deformation of the stamp and substrate during the imprinting.

The dramatic increase in pattern fidelity with the use of porous stamps can be explained by existing literature on (*i*) MACE and (*ii*) diffusion through porous networks. When Chartier et al. (32) performed the etch rate measurement as a function of the ρ-parameter, it was performed with nanoparticles with sizes that were in the 10- to 30-nm range. At this length scale, the diffusion pathway to the center of the catalyst–silicon interface is short, and diffusion toward the catalyst–silicon interface is presumably not the rate-limiting step. Thus, the argument that, at ρ = 75%, the etch rate is maximized due to the stoichiometry balancing of the proposed reaction mechanism holds true. However, Geyer et al. (33) found that—for larger features sizes (i.e., >500 nm) and thicker thin-film catalyst (i.e., >30 nm)—the etch rates were significantly lowered, and a porous silicon layer was formed underneath and around the catalyst to support mass transport of reacting species to and from the center of the features. These results support the idea that diffusion of reacting species becomes the limiting rate step when the diffusion pathway is sufficiently large or the catalyst film does not allow for diffusion through its thickness. In fact, the attained high pattern fidelity over nano- (Fig. 2*D*) and microscales (Fig. 3*D*) with the use of highly mesoporous stamps establishes that the diffusion of reactants and products to the contact interface between stamp and substrate is abundant (31) and that etching is localized to within one order of magnitude of the Debye length (∼0.5 nm) (46). This remains true whether the diffusion pathway is located in the substrate as previously shown in Mac-Imprint of porous silicon (31) or in the porous stamp as shown in this work.

**Fig. 5.**
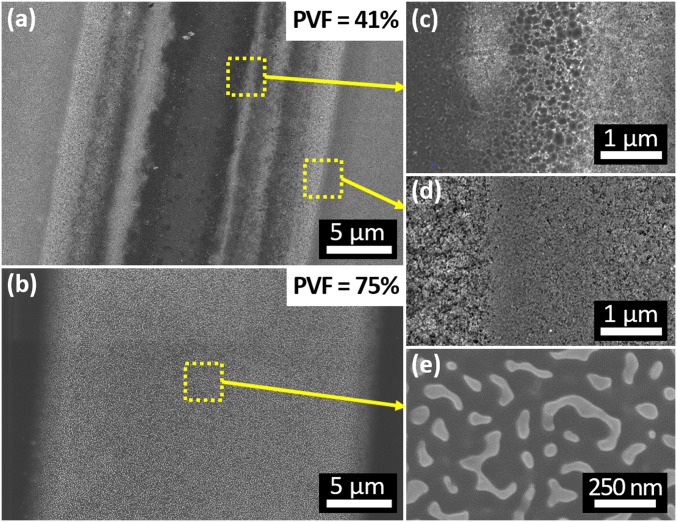
The morphology of imprinted silicon is depicted when using identical stamps and processing conditions, with the only varying parameter being the porosity of the catalyst layer. SEM images of silicon imprinted with porous gold films containing PVF of (*A*) 41 and (*B*) 75% are presented. *C–E* correspond to magnified images in the yellow dashed areas, highlighting the porosity in the center and edge of the imprinted area.

**Fig. 6.**
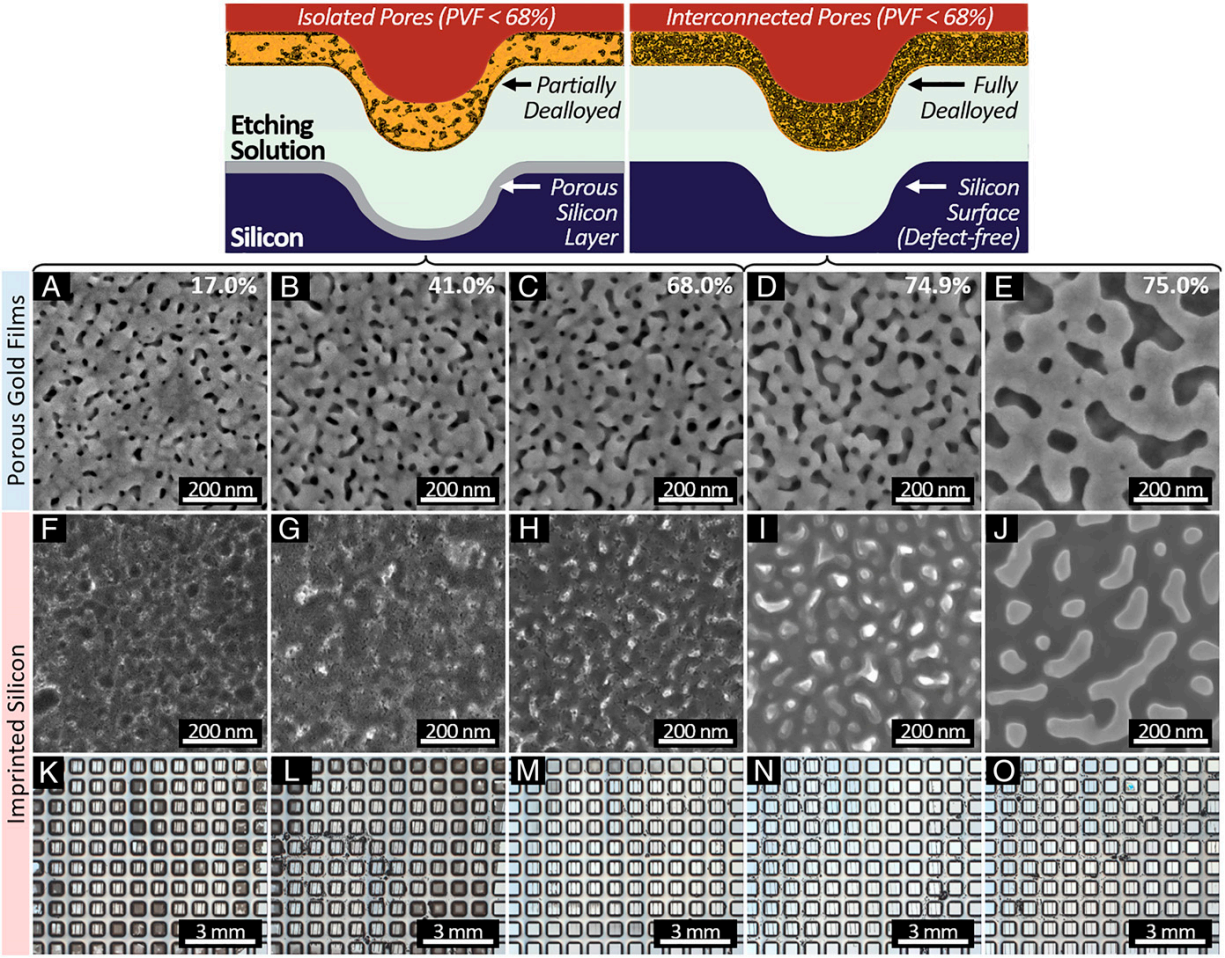
Along the top, the schematics highlight the isolated and interconnected pores in partially porous catalysts and fully porous catalysts, respectively, and the resulting imprinted silicon morphology. In *A*–*O*, a comparison of stamp and substrate morphology via top-down SEM and optical images is presented. *A*–*E* show the detailed morphology of partially porous catalyst films, and in the top-right corner of each image, the apparent PVF is noted. *F*–*J* depict the morphology of the silicon imprinted with the porous catalysts corresponding to its column item above it. *K*–*O* show the imprinted silicon image via optical microscopy; darkening of the substrate is characteristic of porous silicon formation.

Despite the high patterning fidelity, it is not necessarily true that silicon remains intact, since porous silicon may be generated concomitantly during imprinting (30–32, 34), which would result in the creation of a diffusion pathway through the substrate. Thus, we examined the morphology of the imprinted silicon substrate with SEM as a function of a wide range of catalyst porosity and etching time. Partially porosified stamps with a wide range of apparent PVF from 17 to 75% were manufactured by timed dealloying (40). In the case of low stamp porosity (e.g., PVF < 68%), the electrochemical reaction is highly unlocalized, leading to the formation of mesoporous silicon during imprinting surrounding and at the center of the imprinted feature (Fig. 5 *A*, *C*, and *D*). In contrast, highly porous stamps (e.g., PVF = 75%) yield localized etching of the substrate, with no evidence of porous silicon formation near the imprinted area (Fig. 5 *B* and *E*). A sharp decay in the rate of porous silicon formation during imprinting takes place when PVF reaches 68% (Fig. 6 *F*–*H*), and sub–15-nm features from the mesoporous stamp appear on the substrate (as shown in Fig. 6 *I* and *J*). This trend can also be observed in the optical reflectivity of the silicon substrates shown in Fig. 6 *K*–*O* and *SI Appendix*, Fig. S2, since the presence of mesoporous silicon renders the substrate appearance dark under white light illumination. This observed transition from delocalized to localized etching as the stamp becomes highly porous (i.e., PVF > 68%) is analogous to the observations of Geyer et al. (33) of a decrease in porous silicon formation upon an increase of the diffusion rate through thinner and narrower catalyst geometries. Furthermore, this transition from porous silicon formation to anisotropic etching takes place when the diffusion of the reactants and by-products of the anodic reaction toward and away from the silicon–catalyst interface can keep pace with the rate of the cathode reaction, and it has been extensively discussed in a topical review (28). When diffusion through the catalyst is limited, the electron holes in silicon injected by the cathodic reaction diffuse and migrate away from the catalyst–silicon interface, generating porous silicon in the vicinity of the feature (31). Utilizing mesoporous catalysts in MACE is a way to increase the effective diffusion coefficient of the catalyst thin film, reduce the diffusion pathway, and ultimately, restore the diffusion of reacting species from the edge to the center of the contact interface. That is, because when a contiguous/interconnected pore network is formed in the porous catalyst (Fig. 6*D*), molecules and ions diffuse not only through its grain boundaries and bulk grains but also, through the void phase. According to the random network theory, a continuous network is formed when PVF is ∼50% (47), which varies according to the pore network formation models. Although highly tortuous, this void phase possesses an effective diffusion constant that is orders of magnitude higher than that of grain boundaries (47). This is due to the fact that grain boundaries are narrow (i.e., width is in the length scale of the electrical double-layer thickness), which constrains ion mobility, and thus, possess a lower effective diffusion constant. In mesopores, the molecules weakly interact with the pore walls, since the pore sizes are much larger than the electrical double-layer thickness. As a result, imprinting with mesoporous catalyst promotes diffusion and localized etching, minimizing porous silicon formation and ultimately, leading to well-defined multiscale features with large (>10-µm) and small (<15-nm) sizes in a single imprinting operation. Note that pore coarsening in the porous catalyst (Fig. 6 *A*–*E*) is a well-known phenomenon due to progressive formation of adatoms during dealloying and restructuration of the porous film as the etching evolves (48).

**Fig. 7.**
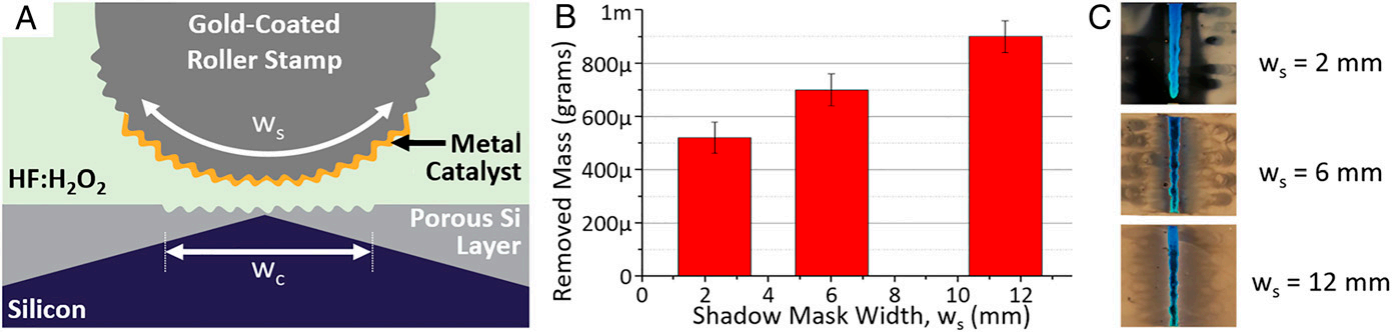
The roller stamps possess an overhanging structure that allows one to vary the catalyst–solution interface area independent of the catalyst–silicon interface area. *A* shows a schematic of the imprinting setup. *B* shows the removed silicon mass obtained from gravimetric analysis as a function of shadow mask or catalyst width (w_s_). *C* shows optical top-down images of the imprinted domain (i.e., approximately 2 cm × 0.2 cm), showing the blue reflection of the imprinted grating line (note that the surroundings are heavily porosified due to the use of solid catalysts). Imprinting time was 10 min.

Unlike thin film-based MACE, in imprinting formats, the ratio of the area of the cathode (i.e., gold surface exposed to MACE solution) and the contact area between the catalyst and silicon (A*) is not restricted to unity, and it can vary depending on the catalyst geometry. This fact raises the question of whether the cathodic reaction (i.e., reduction of hydrogen peroxide) scales with the cathode area. If that, indeed, is true, then the rate of hole injection into silicon can be increased as well, leading to the increase of the rate of porous silicon formation. To test this hypothesis, Mac-Imprint was performed with roller stamps possessing overhanging domains sufficiently large to vary the area of the catalyst relative to the contact area (i.e., A* is equal to w_s_/w_c_ in the configuration shown in Fig. 7*A*) by an order of magnitude. By increasing the overhanging portion of the catalyst (i.e., the portion not in contact with silicon), the cathode area is extended along the catalyst solution interface, while the contact area remains unchanged (as in Fig. 7*A*). After imprinting, gravimetric analysis was used to determine the total mass of silicon removed during imprinting as a function of the shadow mask width (w_s_) for a fixed contact width (w_c_ ∼ 2 mm). It is observed that the total mass of silicon removed scales linearly with the shadow mask width, while the contact width (w_c_) remains constant (Fig. 7*B*). Thus, by keeping the solution parameter (ρ), temperature, time, and pressure constant and only varying the overhanging length of the catalyst, it is demonstrated that the apparent removal rate of silicon increased, which constitutes evidence in the MACE literature that the surface area of the catalyst regulates the kinetics of hole injection. Since the imprinted volume is negligible (i.e., feature depth is ∼300 nm) and the catalyst is solid—and thus, cannot support diffusion—most of the mass removed is attributed to porous silicon formation in the surroundings of the imprinted grating, which is consistent with the observations of the color changes in the vicinity of the imprinted area (Fig. 7*C*).

In future work, this technique requires efforts to develop (*i*) durable stamp materials that can withstand hundreds to tens of thousands of imprinting cycles, (*ii*) novel stamp fabrication approaches that include chemical storage domains embedded onto the stamp rather than on the substrate, (*iii*) modeling of diffusion and reaction kinetics of MACE, (*iv*) in situ process monitoring and metrology strategies, and (*v*) novel stamp preparation methods that can reduce the line-width resolution of the process, such as the use of microporous materials (with sub–2-nm pore sizes) or straight-walled and patterned pores. Thus, the development of novel advanced stamp materials is highly encouraged to enable commercialization of this technique.

In summary, Mac-Imprint has been extended to silicon wafers with the use of mesoporous catalysts. It has been shown that catalyst geometry and porosity are relevant factors to regulate the diffusion pathway, the effective diffusion constant of the porous metal catalyst, and reaction kinetics. First, it was established that stamps with higher apparent PVF play a critical role in enabling diffusion of chemical species during imprinting, which in turn, allows for morphology control of imprinted silicon with features as small as sub-15 nm. Second, the relative area ratio of catalyst–solution to the catalyst–silicon interface plays an important role in regulating the rate of hole injection and consequently, the morphology of imprinted silicon substrates. When combined, these factors capture the considerations that one must have when using Mac-Imprint for generating pristine features into silicon and potentially, other semiconductors. When properly designed, Mac-Imprint produces pristine nano- and microscale features in silicon, allowing for fast replication of silicon patterns from a polymeric mold.

## Methods

First, mesoporous Ag-Au thin films were synthesized by dealloying (48). Second, silicon substrates were prepatterned for subsequent imprinting. Third, porous catalysts were used during imprinting, and the resulting silicon substrate morphology was characterized via SEM and AFM. Fourth, a special stamp preparation and imprinting setup was developed to vary the catalyst overhanging area independent of the contact area.

### Stamp and Substrate Preparation for Mac-Imprint.

The method starts by spinning a 3-µm-thick layer of AZ1518 photoresist supplied by MicroChemicals onto a 4-inch (100) silicon wafer. This layer was baked at 170 °C for 20 min. Next, a second layer was spun and patterned by lithography, and it was baked at 170 °C for 20 min. The mask used contained an array of lines with 10-μm width and 128-μm spacing. After the second baking step, the lines dewet into parabolic half-cylinders. Then, stamps were cosputtered with Ag and Au in an AJA Sputtering System calibrated with a crystal monitor. The deposition pressure was 3 mtorr, the film thickness was 400 nm, and the Ar flow rate was 4.5 sccm. The power values in the Ag and Au targets were set to 95 and 16 W, respectively, and the corresponding sputtering rates were measured to be 3.5 and 1.2 Å/s, respectively. The volume fraction of silver is 0.75. Finally, the wafer was cleaved into 1 × 1-cm chips and partially dealloyed in a solution of nitric acid (70% diluted in water) and deionized water (DI) water mixed at 1:2 ratio; it was kept at 60 °C ± 1 °C with a hot plate and constant stirring for 10, 30, 90, 270, and 810 s. Additional details regarding this step are in *SI Appendix*, Fig. S7 (40). For the planar stamp displayed in Fig. 4, it was produced from a mold made by e-beam lithography on hydrogen silsesquioxane (HSQ) resist patterned with an array of holes with 1-µm width and 2-µm spacing. This mold was transferred to polydimethylsiloxane (PDMS) following the procedure in the literature (49). The PDMS replica was imprinted onto an SU-8 thin film (3-µm thick) spun onto a silicon wafer pressed together by a free weight of 16.15 g for an imprinted area of 0.6 × 0.6 cm and irradiated with a 6-W lamp, which was placed 10 cm away from their interface for 2.5 h. Stamps made in this fashion were also sputtered with the catalyst film and dealloyed for 270 s as described in the previous paragraph. For the Si substrates (p type and with resistivity of 1–10 ohm⋅cm), an array of square pillars was patterned using photolithography followed by deep-reactive ion etching (DRIE) (35) to have enough etchant in the vicinity of the reaction front (31). The pillars had a width of 400 μm, a period of 900 μm, and a height of 60 μm. Next, silicon substrates are cleaned with standard RCA-1 cleaning solution and considered ready for imprinting.

### Imprinting Setup.

Silicon substrates were imprinted with porous stamps under identical conditions. The substrate was immersed in an HF-H_2_O_2_ solution, ρ, of 98% as defined in the literature (32). The stamp mounted to a Teflon holder is brought into contact with the substrate using a servo-controlled motion stage with a load cell until a load of 4 lbf is developed. The two are held together for 1 min (unless otherwise noted), at which point the stamp is withdrawn and the solution is removed. The stamp and substrate are immediately rinsed in DI for a few seconds and air dried. Porous noble metal stamps were reutilized four times for imprinting without any visible damage (such as film peeling or scratching) to the stamp during an inspection in a regular optical microscope (Fig. 3*B*).

### Varying Catalyst Surface Area.

Varying the catalyst surface area was accomplished by sputter coating pieces of a polyethylene holographic grating with a Cr and Au layer (with 10- and 100-nm thickness, respectively) through a shadow mask possessing varying widths (i.e., w_s_) of 2, 6, and 12 mm. Note that the catalyst film is not porous in this experiment. The grating sheet was purchased from Edmund Optics and cut into 1 × 2-cm pieces that were precleaned with isopropyl alcohol (IPA) and DI water. The grating had a nearly sinusoidal cross-section with a constant pitch of 1 μm and an amplitude of 350 ± 50 nm after an inspection with AFM (30). Next, the coated grating piece was wrapped around a Teflon rod 1 cm in diameter and 2 cm in length with Kapton tape and loaded onto a manual *Z* stage. A p-type silicon wafer with 1–10 ohm⋅cm resistivity was degreased and cleaned with the RCA-1 procedure. Then, it was cleaved into a 2-inch^2^ piece and placed onto the surface of a Teflon reservoir. The reservoir was partially filled with a mixture of HF and H_2_O_2_ (at ρ = 75%) such that the substrate was submerged into solution. The load was set to 9 ± 0.5 N during imprinting for 10 min, and samples were rinsed in DI water for 3 min afterward. The average contact width was 2 mm (w_c_).

## Supplementary Material

Supplementary File
