# Supplementary material for: Electrochemical nanoimprinting of silicon
Source: Proc Natl Acad Sci U S A. 2019 May 8;116(21):10264–9. doi: 10.1073/pnas.1820420116 (PMC6535012; doi:10.1073/pnas.1820420116)
Supplement: Supplementary File [file pnas.1820420116.sapp.pdf]

PNAS
www.pnas.org

Supplementary Information for

Electrochemical Nanoimprinting of Silicon

Aliaksandr Sharstniou, Stanislau Niauzorau, Placid Ferreira, Bruno Azeredo*

Bruno Azeredo
Email: bruno.azeredo@asu.edu

This PDF file includes:

Supplementary text
Figs. S1 to S7
Tables S1 to S1

www.pnas.org/cgi/doi/10.1073/pnas.1820420116

SUPPLEMENTARY INFORMATION

In this supplementary section, more information about the experimental results of silicon imprinting with porous stamps is presented.

1.1 Description of sample inspection and data collection

The imprinted feature topology was measured with AFM at selected locations. By overlaying optical and AFM results, it is possible to distinguish the imprinted domains (i.e. where the catalyst was in contact with the silicon substrate) from the porous silicon formation surrounding the features (Supplementary Figure 1). Additional details regarding sample observations are also available in previous publication [1].

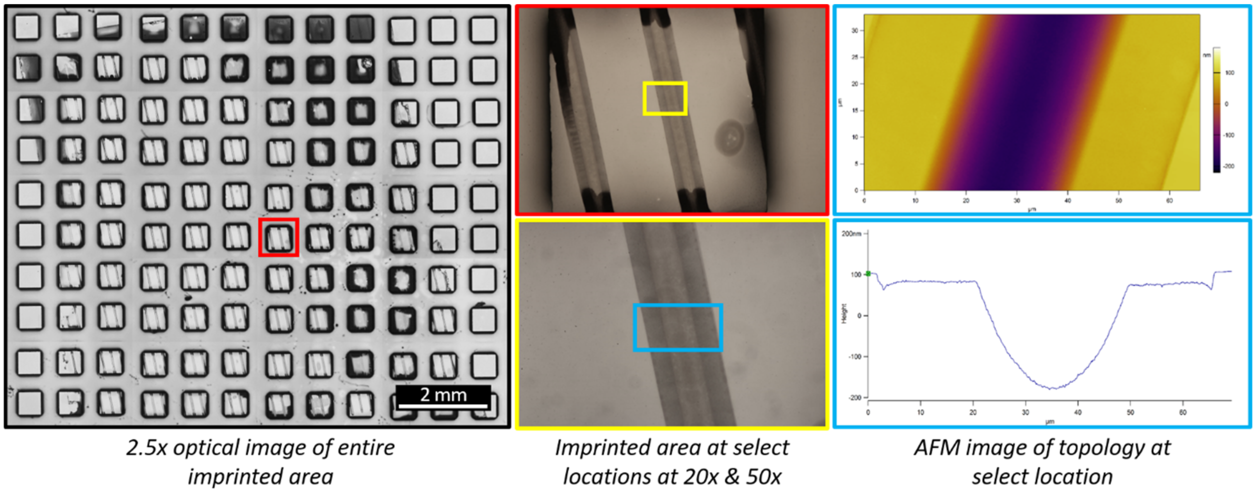

Supplementary Figure 1: Description of method of data collection of imprinted silicon substrates [1].

1.2 Further details on imprinting with porous gold catalyst

First, the entire imprinted domains (i.e. approximately 1 cm^2) were imaged with an optical microscope at 2.5x and at select locations near the center of the imprinted domain at 20x and 50x (see Supplementary Figure 1). On discussion related to Figure 6 of the paper, it was stated that imprinting with low-PVF (i.e. 17-68%) leads to delocalized etching and

porous silicon formation and, as the stamp becomes more porous (i.e. 68-75%), the etching is localized to the stamp-silicon contact interface. Additional evidence of this phenomena is presented in Supplementary Figure 2 where the 2.5x optical images of the imprinted silicon substrates are shown and darkening of the substrate – which is indicative of porous silicon formation - was observed for low-PVF values (Supplementary Figure 3) and long etching times (Supplementary Figure 4).

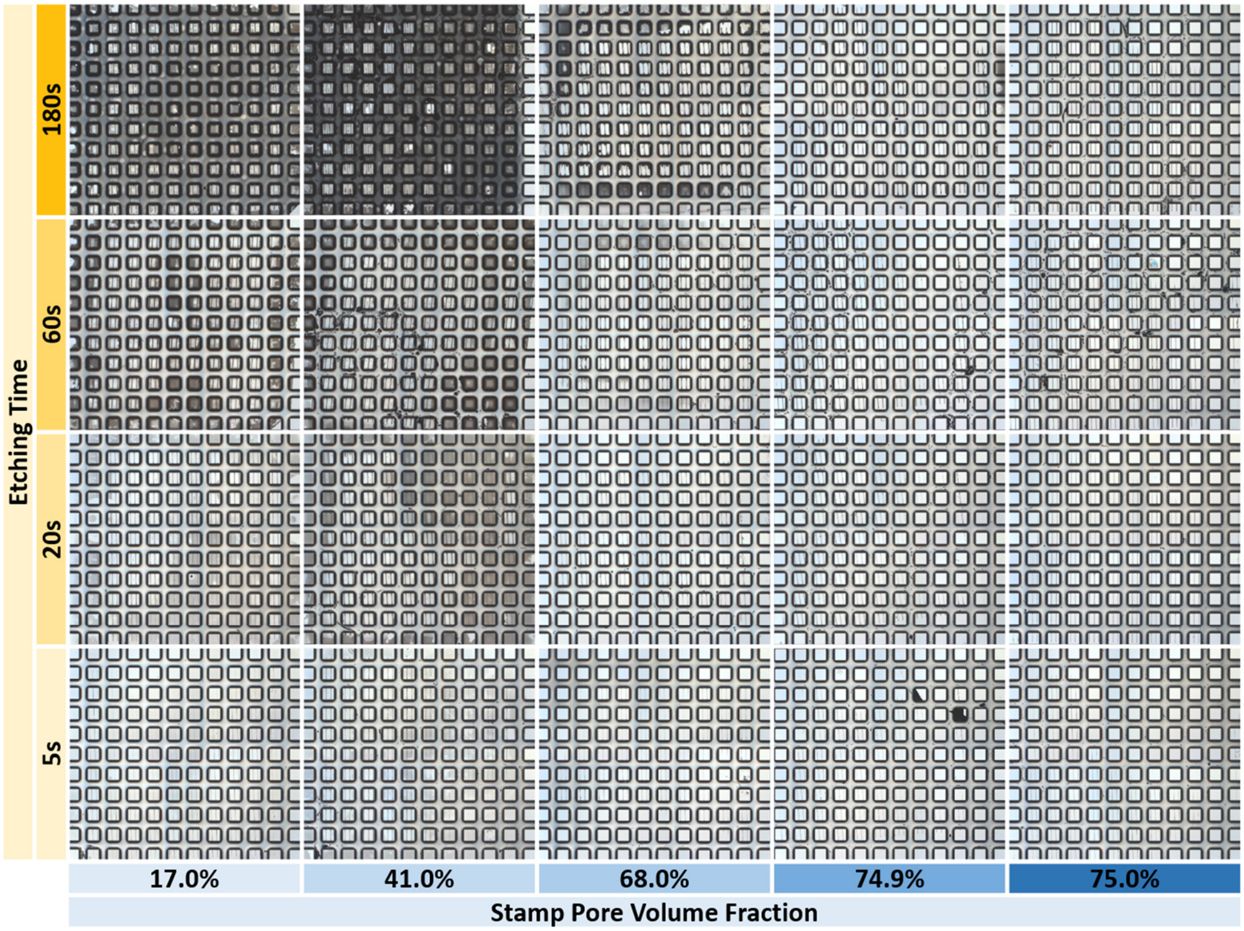

Supplementary Figure 2: Optical images of entire 1 cm x 1cm domains with a 2.5x magnification with top-down illumination. Images are arranged as a function of the imprinting time and the PVF of the stamp used to imprint. All images are taken at the same light exposure conditions without contrast or brightness adjustments. Note that each row of samples is patterned with the same stamp and that the patterning area is roughly constant suggesting that stamps do not degrade significantly.

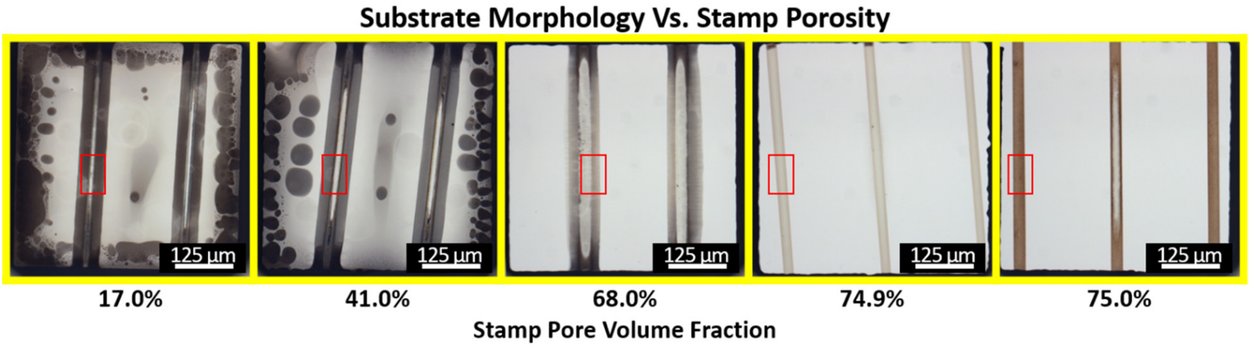

Supplementary Figure 3: Optical image at 20x magnification of imprinted silicon substrates etched under ρ=98% and for 60s. Near vertical lines correspond to contact areas between stamp and substrate. Porous silicon domains form in the surroundings of the imprinted features. At lower PVF, porosification of the substrate is evidenced by the darkening of the silicon surface. All images are taken at the same light exposure conditions without contrast or brightness adjustments.

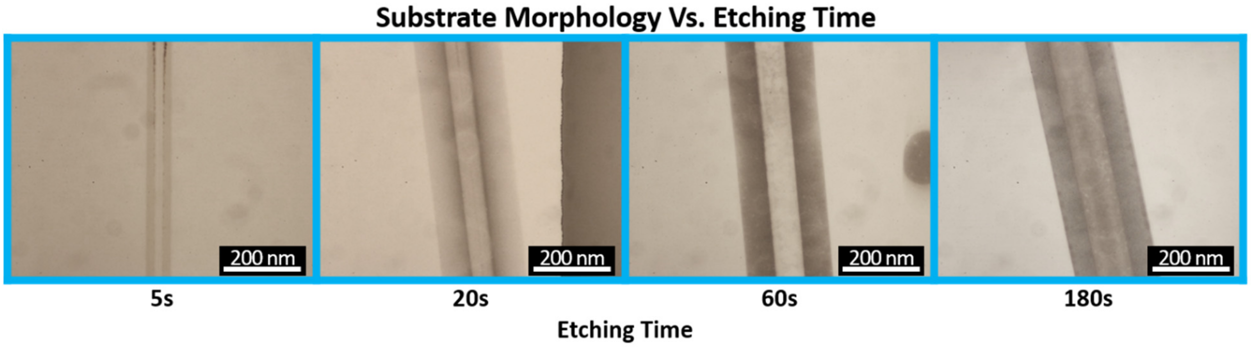

Supplementary Figure 4: Optical images at 50x magnification of imprinted silicon substrates for different etch times at ρ=98% and PVF=68%. All images are taken at the same light exposure conditions without contrast or brightness adjustments.

Top-down SEM images of the features at selected locations were collected. In the case of PVF =41%, the images reveal the mesoporous nature of the silicon surface near the imprinted domain and in its surroundings (Supplementary Figure 5). In the case PVF=75%, the SEM images do not reveal any mesoporous silicon and patterns are well defined resembling the features of the stamp (Supplementary Figure 6).

41.0%

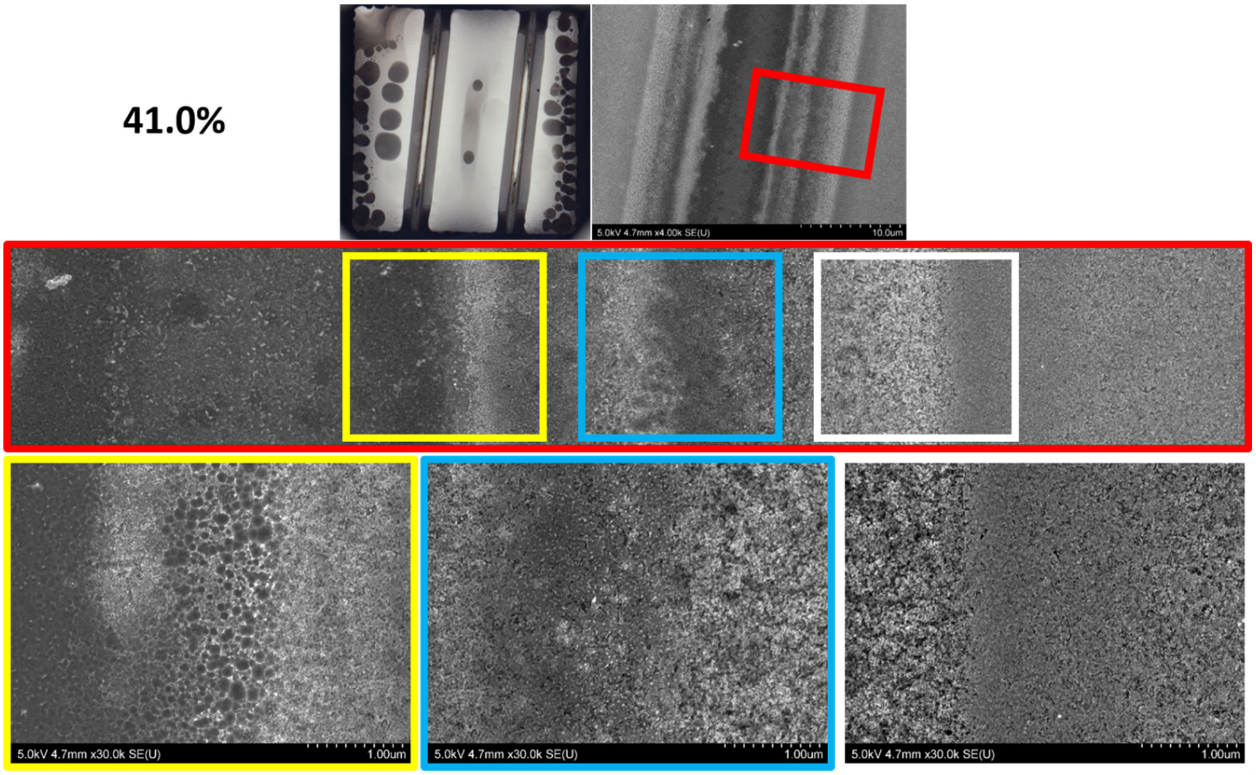

Supplementary Figure 5: At the top row, the optical image (on the left) shows the selected 500 µm x 500 µm domain of the silicon substrate. At that location, SEM images taken near the imprint area reveal the porous nature of the substrate from the center and towards the edge of the imprinted domains. Clearly, patterning has no fidelity in this case and pores are generated at the contact interface and on its surroundings.

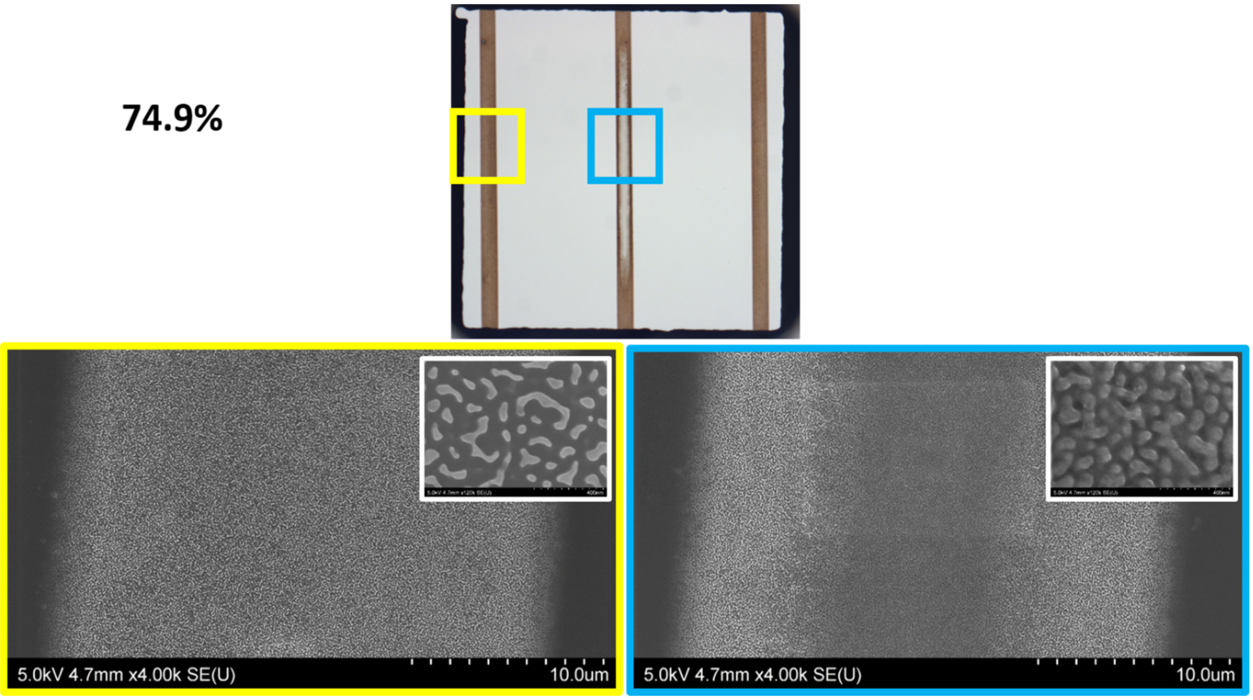

Supplementary Figure 6: At the top row, the optical image (on the left) shows the selected 500 µm x 500 µm domain of the silicon substrate. At that location, SEM images taken near the imprint area reveal the porous nature of the substrate from the center and towards the edge of the imprinted domains. Clearly, patterning has no fidelity in this case and pores are generated at the contact interface and on its surroundings.

1.3 Further details on analysis of stamp porosity

In order to extract the apparent pore volume fraction as a function of dealloying time, it was first necessary to analyze the mass data shown in Supplementary Figure 7b. Since samples are cleaved into pieces that are not of identical dimensions (Supplementary Figure 7a), the data was analyzed in a scheme that eliminates the need for area measurements of each sample and the introduction of additional errors. Rather than using the difference of the initial and final mass which is dependent on sample size, it was chosen to compare the mass ratio because, as shown below, it is independent of the sample size. Next, a formula for the mass ratio is derived. First, the initial mass (M_o) of the samples is defined as the sum of the products of the density (ρ_i), area (A_i) and thickness (t_i) of each layer (i.e. silicon wafer, photoresist, chromium and Au-Ag thin film) and it can be expressed as:

$$M_o = \left[\sum_{i=Si,PR,\,Cr,\,Au} \rho_i (At_i) \right] + \rho_{AgAu}(At_{AgAu})$$

The density of the thin-film alloy can also be expressed as a function of the initial volume fraction of silver (V_o) as follows:

$$\rho_{AgAu} = \rho_{Ag}(V_o) + \rho_{Au}(1 - V_o)$$

Assuming that only silver atoms are etched during the dealloying step, the final mass of the samples (M_f) can thus be written as a function of the final volume fraction of silver in the alloy film (V_f):

$$M_f = \left[\sum_{i=Si,PR,\,Cr,\,Au} \rho_i (At_i) \right] + \left[\rho_{Ag}(V_f) + \rho_{Au}(1 - V_o)\right] At_{AgAu}$$

As a result, the mass ratio can be simplified to the equation below:

$$Mass\ Ratio = \frac{M_o - M_f}{M_o} = C_o\left(V_o - V_f(t)\right)$$

$$where\ C_o = \frac{\rho_{Ag} t_{AgAu}}{\sum_{i=Si,PR,\,Cr,\,Au} \rho_i(t_i) + \rho_{AgAu}(t_{AgAu})}$$

Note that this equation becomes independent of the area of the sample and C_o becomes a fixed parameter dependent only on the initial characteristics of the film. Also, the difference V_o-V_f is the apparent pore volume fraction (i.e. volume of pores divided by the initial volume of the film), which neglects morphological changes of the thin-film during

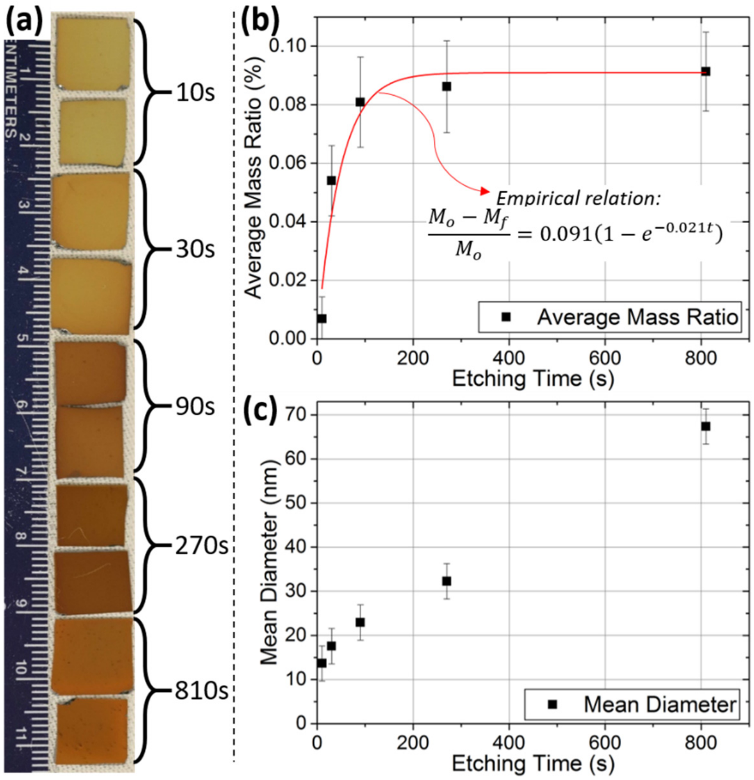

Supplementary Figure 7: In part (a), porous stamps, cut into approximately 1x1 cm squares, are depicted in the photograph after dealloying for a specific etching time (denoted in the image). Part (b) and (c) are plots of the mass ratio and mean pore diameter of dealloyed Ag/Au films as a function of etching time obtained from gravimetric analysis and image analysis of SEM data, respectively.

dealloying (such as shrinking). Next, the following empirical formula, in which C_o and k are fitting parameters, was used to fit the mass ratio data in Supplementary Figure 7b. This formula was selected not only because it fits well the data, but also because it converges to a fixed and maximum value (i.e. V_o) which agrees well with the notion that silver is completely removed at sufficiently long etch times:

$$\frac{M_o - M_f}{M_o} = C_o V_o (1 - e^{-kt})$$

Thus, the apparent pore volume fraction can be extracted from the curve fitting and is recorded in Table 1.

Table 1: etching time correlation with apparent pore volume fraction

Time (s)	10	30	90	270	810
Apparent Pore Volume Fraction	17.4%	41.0%	68.0%	74.9%	75.0%

References

[1] Aliaksandr Sharstniou; Stanislau Niauzorau; Bruno Azeredo, "Electrochemical Nanoimprinting Of Silicon: A Direct Patterning Approach," In Spie - Advanced Lithography, San Jose, California, United States, 2018.
